# Anti-obesity effect and mechanism of mesenchymal stem cells influence on obese mice

**DOI:** 10.1515/biol-2021-0061

**Published:** 2021-06-25

**Authors:** Zongyan Xie, Yu Cheng, Qi Zhang, Haojie Hao, Yaqi Yin, Li Zang, Xuhong Wang, Yiming Mu

**Affiliations:** Department of Clinical Pharmacology, Beijing Luhe Hospital Affiliated to Capital Medical University, 82 Xinhua South Road, Beijing 101149, People’s Republic of China; Department of Endocrinology, The First Medical Center of PLA General Hospital, 28 Fuxing Road, Beijing 100853, People’s Republic of China; Department of Endocrinology, Beijing Tiantan Hospital Affiliated to Capital Medical University, Beijing 100070, People’s Republic of China; Department of Molecular Biology, Institute of Basic Medicine, The First Medical Center of PLA General Hospital, Beijing 100853, People’s Republic of China

**Keywords:** adipose tissue, mesenchymal stem cell, energy expenditure, obesity, macrophage

## Abstract

Mesenchymal stem cells (MSCs) can be obtained from almost all tissues and present promising therapeutic effects for metabolic diseases. Human adipose-derived MSCs (hASCs) have recently been widely studied due to their easy access and low immunity. Thus, we intended to figure out the effects and potential mechanism of hASCs on obesity in high-fat-diet (HFD)-induced obese mice. Following 16 weeks of being fed HFD, hASCs were intravenously injected. Two weeks later, body weight, body composition, and energy expenditure were evaluated. Additionally, the phenotypes of macrophages infiltrating adipose tissue were analyzed. The results revealed that hASCs administration significantly reduced adipose tissue weight, adipocyte size, and fat mass and exerted beneficial effects in serum lipid profile. This anti-obesity effect was mediated by the increased O_2_ consumption, CO_2_ production, and energy expenditure, which was further evidenced by the upregulation of uncoupling protein-1 (UCP-1) and metabolism-associated genes. Furthermore, hASCs infusion increased the amount of alternatively activated (M2) macrophages in adipose tissue, and the expression of pro-inflammatory cytokines-related genes was reduced. Taken together, these results indicated that hASCs suppressed obesity by increasing UCP-1 expression and enhancing energy expenditure, and this effect might be due to the increased M2 macrophages.

## 1 Introduction

Obesity has increased at an alarming rate during the few decades throughout the world [1,2,3,4] and has been regarded as a significant risk factor for hepatosteatosis [5], diabetes [6], cancer [7], etc. Therefore, the high incidence of obesity and its associated health problems alerts us that effective interventions are urgently needed to restrict obesity. Nowadays, several therapeutic options such as lifestyle modification, medications, and surgery are feasible for obesity treatment. However, lifestyle modification has limited effects, most medications are withdrawn because of adverse effects, and surgery is invasive and remains contentious to long-term efficacy and safety [8]. Thus, seeking a new treatment that can overcome previous limitations is very attractive for researchers.

According to their phenotypes and functions, adipose tissues can be categorized into white adipose tissue (WAT) and brown adipose tissue (BAT). WAT is involved in fat storage and contains white adipocytes with unilocular lipid droplets [9,10]. The major cell type present in BAT is brown adipocyte, characterized by multichambered lipid droplets and high density of mitochondrial with highly expressed uncoupling protein-1 (UCP-1). Previous studies have revealed that BAT dissipates excessive energy into heat via UCP-1 and protects against obesity and its related disorders [11,12]. Thus, promoting the expression of UCP-1 and enhancing energy expenditure could be a promising approach to restrict obesity. Recently, accumulated studies have indicated that bariatric surgery [13], exercise [14], and cold stimulation [15] could facilitate WAT browning and limit obesity. Further mechanism research has demonstrated that alternatively activated (M2) macrophages related to catecholamine are crucial for adipose tissue browning [15,16]. Moreover, methods aimed to increase M2 macrophages in adipose tissue have been demonstrated to be effective in limiting obesity [16,17,18].

Mesenchymal stem cells (MSCs) are fibroblast-like stem cells characterized by exceptional self-renewal capacity and differential potential to various cell types. It is generally known that MSCs can be widely obtained from various adult tissues and can expand rapidly *in vitro*. Therefore, owing to its easy access and low immunity, the therapeutic effect of MSCs has acquired much more attention [19]. Till now, researchers have suggested that MSCs may display their therapeutic effect through immunomodulation, which is directed by eliciting M2 macrophages [20,21]. Intravenously infused MSCs have been confirmed to promote M2 polarization in different disease models such as renal ischemia-reperfusion injury [22], acute myocardial infarction [23], and corneal epithelial wound healing in diabetic mice [24]. Furthermore, our study group has demonstrated that MSCs infusion promoted M2 polarization, reduced inflammation, and eventually alleviated insulin resistance in type 2 diabetic rats and mice [25,26,27]. As mentioned, MSCs have been verified to induce M2 macrophages, and M2 macrophages have been confirmed to be effective in combatting obesity. Therefore, we hypothesized that MSCs infusion could limit obesity by inducing M2 macrophages.

Adipose-derived mesenchymal stem cells (ASCs) are originated from the stromal vascular fraction (SVF) of adipose tissues. Compared to other MSCs, ASCs are considered superior because adipose tissue can be acquired easily and repetitively. The isolation procedure is rather simple, less invasive, and provides a significantly higher concentration of isolated cells [28]. Thus, in this study we transplanted human adipose-derived mesenchymal stem cells (hASCs) into high-fat-diet (HFD)-induced obese mice via the tail vein to investigate the anti-obesity effect of hASCs. Additionally, we aimed to clarify the potential mechanism of hASCs in terms of energy metabolism.

## 2 Materials and methods

### 2.1 Isolation, culture, and identification of hASCs

Human adipose tissue was freshly obtained from the abdominal wall of a simple obese patient (35 years old, male, BMI = 35.1) who underwent liposuction at the First Medical Center of the PLA General Hospital. Adipose tissue was washed thoroughly with phosphate-buffered saline (PBS) and cut into pieces smaller than 1 mm^3^. The adipose tissue was digested with low glucose Dulbecco’s modified Eagle’s medium (DMEM) (Gibco, USA) containing 0.05% trypsin and 0.1% type 1 collagenase at 37°C for 40 min. Digestion was ended by the addition of 10% fetal bovine serum (FBS) (Gibco, USA). Then, the floating adipocytes were removed by filtration through a 100 µm metal mesh. Then, SVF was isolated from centrifugation at 1,000 rpm for 5 min and resuspended in low glucose DMEM with 100 U/mL penicillin–streptomycin and 10% FBS and incubated at 37°C, 5% CO_2_, and 95% humidity. The next day, floating cells were taken out by changing the medium. Then, we changed the medium in 2–3 days. After cell fusion, the cells were passaged at a ratio of 1:3. The 4th generation cells were obtained for our study. Adipogenic and osteogenic differentiation was performed using the cell differentiation kit from R&D Systems (Minneapolis, MN, USA) to determine the pluripotent differentiation traits of hASCs. hASCs (1 × 10^6^) at passage 4 were collected, washed twice with PBS, and then incubated with antibodies against human CD34 (1:50; cat. no. 550761; BD Biosciences, Inc.), CD45 (1:50; cat. no. 555482; BD Biosciences, Inc.), CD90 (1:50; cat. no. 555595; BD Biosciences, Inc.), CD73 (1:50; cat. no. 550257; BD Biosciences, Inc.), CD105 (1:50; cat. no. 560839; BD Biosciences, Inc.), and HLA-DR (1:50; cat. no. 555560; BD Biosciences, Inc.). Phenotype identification was analyzed by flow cytometer (Becton Dickinson, Inc.).


**Informed consent:** Informed consent has been obtained from all individuals included in this study.
**Ethical approval:** The research related to human use has been complied with all the relevant national regulations, institutional policies, and in accordance with the tenets of the Helsinki Declaration and has been approved by the Medical Ethics Committee of PLA General Hospital (approval no. S2013-107-01).

### 2.2 Animal experiments

Eight-week-old male C57Bl/6 mice (weight 17–18 g) were obtained from PLA General Hospital. They were housed under a standard environment (room temperature of 22 ± 1°C, humidity of 55 ± 5%, 12 h light/dark cycle) and allowed to eat and drink freely. After one week of adaptation, mice were randomly administered HFD (cat. no. D12492, %kcal; carbohydrate: protein: fat = 20:20:60, Research Diets) (cat. no. D12492, %kcal), with fat consisting of soybean oil and lard (soybean oil: lard = 1:9.8), which indicated that the main fat component was saturated fat to induce obese mice (*n* = 12) or normal chow diet (NCD) to induce normal control group (*n* = 6). The body weight of each mouse was measured once a week, and total food consumption was recorded daily. Sixteen weeks later, obese mice were randomized to a single intravenous infusion of 1 × 10^6^ hASCs suspended in 0.2 mL PBS via the tail vein (the hASC group, *n* = 6) or 0.2 mL PBS alone (the obese group, *n* = 6). Two weeks later, calorimetry and EchoMRI experiments were performed on the mice. Afterwards, mice were fasted for 12 h and then sacrificed. Blood was immediately collected for lipid metabolism analysis through the intraorbital vein. Interscapular BAT, inguinal subcutaneous adipose tissue (ingWAT), and epidermal adipose tissue (epiWAT) were collected and measured. Adipose tissues were stored in liquid nitrogen for mRNA/protein analysis and fixed in formalin for histological analysis.


**Ethical approval:** The research related to animal use has been complied with all the relevant national regulations and institutional policies for the care and use of animals and was approved by the Institutional Animal Care and Use Committee of PLA General Hospital (approval no. 2014-H121-5).

### 2.3 Lipid metabolism analysis

Blood samples were centrifuged at 1,000 rpm for 10 min and the serum was separated. 200 µL plasma samples were collected for lipid analysis, including low-density lipoprotein cholesterol (LDL-c), triglycerides (TG), high-density lipoprotein cholesterol (HDL-c), and total cholesterol (TC), using an automated biochemical analysis machine (Cobas c701, Roche) with an enzymatic colorimetric assay kit (Roche). All methods were operated based on the instructions of the assay kit.

### 2.4 Indirect calorimetry and body composition analysis

Two weeks after hASCs infusion, mice were housed separately in metabolic cages (Oxylet, PanLab, Spain) and acclimated for 24 h before measuring oxygen (O_2_) consumption and carbon dioxide (CO_2_) production (*n* = 6/group). Mice were placed in individually ventilated cages with a controlled room temperature of 22°C and a 12 h light/dark cycle. All mice were allowed to eat and drink freely. The activity of mice was monitored by activity sensors and food was monitored by food sensors. Food consumption was monitored by food sensors. The respiratory quotient ([RQ] = VCO_2_/VO_2_) was calculated from gas exchange data. Energy expenditure was calculated as EE (kcal/day/kg^0.75^) = ([3.815 + 1.232 × RQ] × VO_2_ × 1.44). All data were automatically recorded and calculated using SMART 3.0 software (Metabolism v2.2). Body composition was immediately analyzed after calorimetric experiments. Mice were placed in a clear plastic holder without anesthesia or sedation, and the EchoMRI device (Echo Medical Systems, USA) was used to measure whole body fat and lean mass. All tests were conducted three times and the mean was calculated.

### 2.5 Histologic analysis

Morphology was studied in BAT, ingWAT, and epiWAT sections stained with hematoxylin–eosin. All tissues were fixed in 10% formalin at room temperature for at least 24 h and embedded in paraffin before cutting 5 µm sections. Subsequently, the sections were stained with hematoxylin for 5 min and eosin for 1 min at room temperature with hematoxylin–eosin staining kit (HE, Richard Allan Scientific, Kalamazoo, MI). The stained sections were observed and photographed using an Olympus BX-50 system (Olympus, Tokyo, Japan). The size of 300 adipocytes per mouse from 6 mice was measured using the ImageJ software program (version 1.45, National Institutes of Health, Bethesda, MD, USA).

### 2.6 Isolation and flow cytometry analysis of SVF

epiWATs were gathered, washed thoroughly with PBS, and then cut into pieces smaller than 1 mm^3^. The tissues were digested with low glucose DMEM containing 0.05% trypsin and 0.1% type 1 collagenase, and then filtered through a 100 µm metal mesh. The detailed method was the same as above. SVF pellets were treated with erythrocyte lysis buffer (BD Biosciences, Inc.) and then incubated for 10 min at room temperature with antibodies against mouse F4/80 (1:20; cat. no. 565410; BD Biosciences, Inc.), CD11c (1:20; cat. no. 553801; BD Biosciences, Inc.), and CD206 (1:20; cat. no. 141708; Biolegend, Inc.). Thereafter, cells were washed, resuspended in wash buffer, and then analyzed by flow cytometry (Becton Dickinson, Inc.).

### 2.7 Western blot analysis

Tissues were homogenized in lysis buffer with protease inhibitors (Sigma, St. Louis, MO, USA). Supernatants were collected for protein quantification through centrifugation at 12,000 *g* for 20 min. Bicinchoninic Acid protein assay kit (Kang Wei, Beijing, China) was used to measure protein concentrations. 20 µg of each protein sample was resolved on a 10% SDS-PAGE gel, and the proteins were electrically transferred to polyvinylidene fluoride membranes (Millipore, Inc.). The membranes were blocked with 10% nonfat milk for 1 hour at room temperature, and then were treated with primary antibodies UCP-1 (1:1,000, cat. no. ab10983, Abcam), inducible nitric oxide synthase (iNOS) (1:1,000, cat. ab49999, Abcam), arginase-1 (Arg1) (1:300, cat. no. sc-18354, Santa Cruz Biotechnology), and β-actin (1:2,500, cat. no. 3700s, Cell Signaling Technology) overnight at 4°C. Then, the membranes were incubated with the secondary antibodies goat anti-rabbit (1:3,000, cat. no. ZB2301, ZSGB-Bio company), goat anti-mouse (1:3,000, cat. no. ZB2305, ZSGB-Bio company), and rabbit anti-goat (1:3,000, cat. no. ZB2306, ZSGB-Bio company) IgG horseradish peroxidase for 2 h at room temperature. Finally, proteins were visualized with chemiluminescent substrates of eECL Western Blot Kit (Kang Wei, Beijing, China) and exposed to film in a dark room. Quantitative analysis of protein density was performed with ImageJ software (version 1.45, National Institutes of Health, Bethesda, MD, USA), using β-actin as a loading control, and the relative amounts of each protein were obtained by the ratio to β-actin.

### 2.8 Reverse transcription-quantitative polymerase chain reaction (RT-qPCR) of adipose tissues

RNA samples from epiWAT were extracted using TRIzol reagent (Invitrogen, Carlsbad, USA) and then reversely transcribed to single-stranded cDNA using a reverse transcriptase kit (Invitrogen, Carlsbad, USA) following the manufacturer’s method. Finally, RT-qPCR was performed in a 7500 Real-Time PCR system using SYBR Green PCR reagents (Applied Biosystems, Carlsbad, USA). The reaction system and program were as follows: the cycling stage 40 cycles with 94℃ for 30 s, then 62℃ for 30 s and 72℃ for 30 s; the melt curve stage is 95℃ for 15 s, 60℃ for 60 s, 95℃ for 30 s, and then 60℃ for 15 s. The single peak dissolution curve showed the specificity of the primers. β-Actin was used as an internal control, and the relative mRNA expression of the genes was calculated by the 2^−ΔΔCt^ method. RNase-free DNase I was used to avoid genomic DNA (gDNA) contamination. RNA quality was determined by measuring the A260/A280 ratio and by agarose gel electrophoresis. A negative group with only RNA sample was included in the qRT-PCR assay to verify that RNA was gDNA-free. Reference gene selection was performed according to publications which used the same model (HFD-induced obese mouse) and analyzed the same tissue (adipose tissue) [16,17,29,30]. In addition, Ct values of beta-actin were stabilized at 12–13 during repeated experiments. The primer sequences were listed in Tables 1 and 2.

**Table 1:** Primer sequences of thermogenic-related genes

Genes	Primer sequence (5′–3′)	*T*_m_ (°C)	Product size (bp)	Amplification efficiency (%)
Th	For: CCAAGGTTCATTGGACGGC	59.12	138	99.5
	Rev: CTCTCCTCGAATACCACAGCC	59.93		
UCP-1	For: GTGAACCCGACAACTTCCGAA	60.81	78	98.2
	Rev: TGCCAGGCAAGCTGAAACTC	61.17		
Cox8b	For: TGTGGGGATCTCAGCCATAGT	60.34	62	96.1
	Rev: AGTGGGCTAAGACCCATCCTG	61.25		
Prdm16	For: CAGCACGGTGAAGCCATTC	59.50	87	96.9
	Rev: GCGTGCATCCGCTTGTG	59.86		
Cidea	For: TGCTCTTCTGTATCGCCCAGT	61.23	113	95.8
	Rev: GCCGTGTTAAGGAATCTGCTG	59.60		
Cpt1a	For: TGGCATCATCACTGGTGTGTT	60.20	134	98.2
	Rev: GTCTAGGGTCCGATTGATCTTTG	58.63		
Acox1	For: GCCCAACTGTGACTTCCATTAA	58.85	101	99.1
	Rev: GTAGCACTCCCCTCGAGTGAT	61.02		
Dio	For: CAGTGTGGTGCACGTCTCCAATC	64.02	131	96.3
	Rev: TGAACCAAAGTTGACCACCAG	58.63		
Acsl1	For: TGGGGTGGAAATCATCAGCC	60.03	285	95.8
	Rev: CACAGCATTACACACTGTACAACGG	62.52		
Tmem26	For: ACCCTGTCATCCCACAGAG	58.31	123	95.7
	Rev: TGTTTGGTGGAGTCCTAAGGTC	59.63		
Tbx1	For: GGCAGGCAGACGAATGTTC	59.20	103	94.9
	Rev: TTGTCATCTACGGGCACAAAG	58.58		
Cd137	For: CGTGCAGAACTCCTGTGATAAC	59.33	104	97.5
	Rev: GTCCACCTATGCTGGAGAAGG	59.86		
Pgc1α	For: AGCCGTGACCACTGACAACGAG	64.95	168	97.9
	Rev: GCTGCATGGTTCTGAGTGCTAAG	62.02		

For, forward; Rev, reverse; Th, tyrosine hydroxylase; UCP-1, uncoupling protein-1; Cox8b, cytochrome c oxidase subunit 8b; Prdm16, PR domain containing 16; Cidea, cell death inducing DFFA like effector a; Cpt1a, Carnitine palmitoyl transferase 1a; Acox1, acyl-CoA oxidase 1; Dio, iodothyronine deiodinase; Acsl1, Long-chain acyl-CoA synthetase 1; Tmem26, transmembrane protein 26; Tbx1, T-box transcription factor 1; Pgc1α, peroxisome proliferator activated receptor γ coactivator 1α.

**Table 2:** Primer sequences of macrophages-related genes

Genes	Primer sequence (5′–3′)	*T*_m_ (°C)	Product size (bp)	Amplification efficiency (%)
β-actin	For: CCAGTTGGTAACAATGCCATGT	59.44	154	99.0
	Rev:GGCTGTATTCCCCTCCATCG	59.96		
CD68	For: CATCAGAGCCCGAGTACAGTCTACC	63.93	97	98.2
	Rev: AATTCTGCGCCATGAATGTCC	59.59		
F4/80	For: CTTTGGCTATGGGCTTCCAGTC	61.27	165	99.1
	Rev: GCAAGGAGGACAGAGTTTATCGTG	61.44		
iNOS	For: ACCTTGGTGAAGGGACTGAG	58.94	102	96.5
	Rev: TCCGTTCTCTTGCAGTTGAC	58.13		
Arg1	For: AGACCACAGTCTGGCAGTTG	59.89	74	95.4
	Rev: CCACCCAAATGACACATAGG	56.09		
CD163	For: GGGTCATTCAGAGGCACACTG	60.95	88	97.2
	Rev: GCTGGCTGTCCTGTCAAGGCT	64.98		
CD206	For: TGATTACGAGCAGTGGAAGC	57.99	126	95.7
	Rev: GTTCACCGTAAGCCCAATTT	56.61		
MCP1	For: AGGTCCCTGTCATGCTTCTG	59.38	167	97.8
	Rev: GCTGCTGGTGATCCTCTTGT	60.04		
TNFα	For: CCAGACCCTCACACTCAGATC	57.14	81	98.1
	Rev: CACTTGGTGGTTTGCTACGAC	54.38		
IL1β	For: TGGGCCTCAAAGGAAAGAAT	57.00	216	96.9
	Rev: CAGGCTTGTGCTCTGCTTGT	60.17		
IL10	For: GCTCTTACTGACTGGCATGAG	58.45	105	97.0
	Rev: CGCAGCTCTAGGAGCATGTG	60.88		

For, forward; Rev, reverse; iNOS, inducible nitric oxide synthase; Arg1, arginase-1; MCP1, monocyte chemotactic protein 1; TNFα, tumor necrosis factor α; IL1β, interleukin 1β; IL10, interleukin 10.

### 2.9 Statistical analysis

In this study, each experiment was conducted at least 3 times. All data were analyzed using SPSS 19.0 software (SPSS Inc., IBM, USA). Data were expressed as mean ± standard deviation (SD). Sample means were compared by unpaired *t*-test or one-way ANOVA. Two-tailed *p* < 0.05 was defined as having statistical significance.

## 3 Results

### 3.1 Identification of hASCs

**Figure 1:**
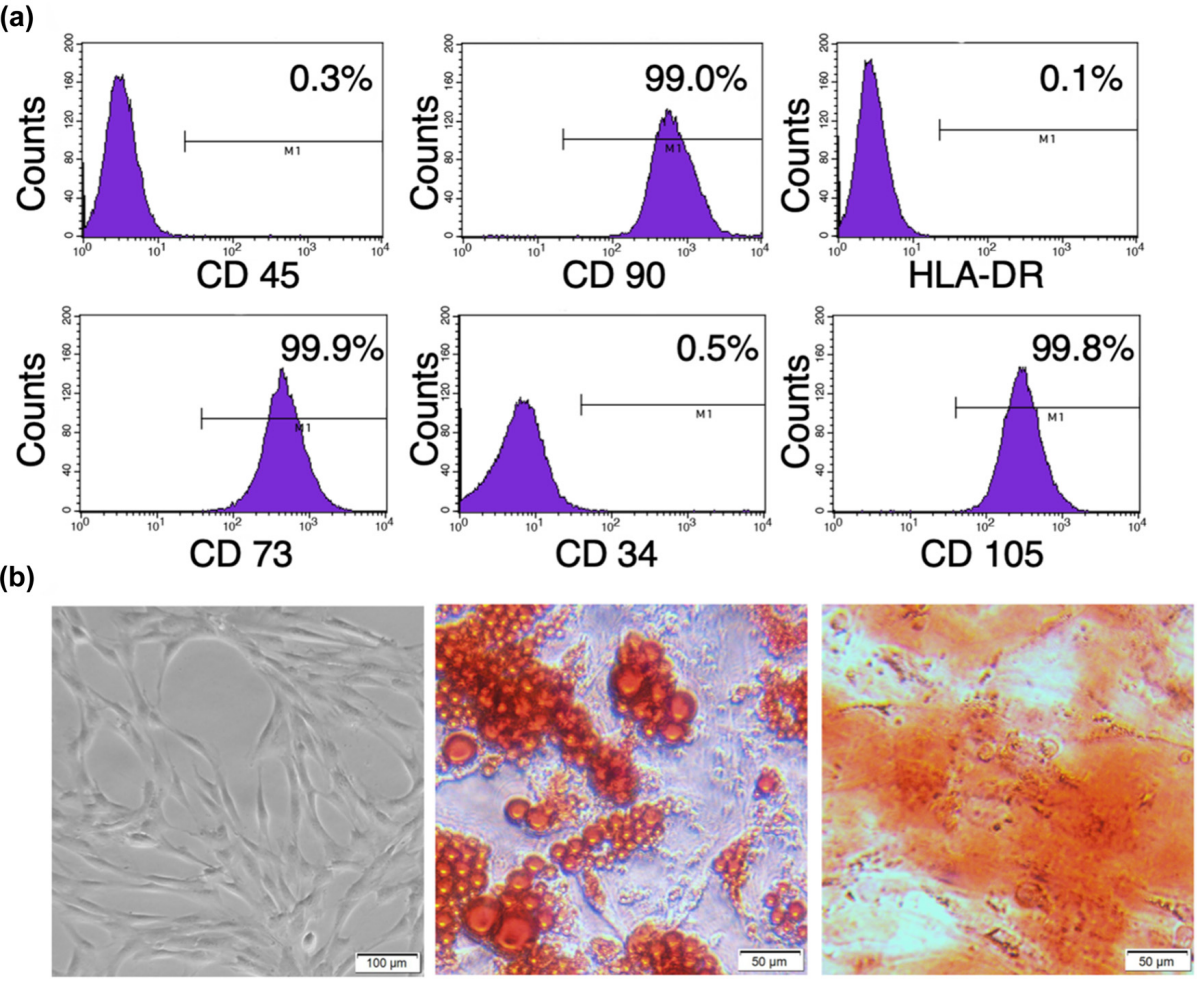
Identification of hASCs. hASCs were identified by their immunologic phenotypes and potential to differentiate into adipocytes and osteoblasts. (a) hASCs were positive for CD90, CD73, and CD105, and negative for CD34, CD45, and HLA-DR. (b) The morphology of hASCs in high magnification (scale bar is 100 µm, left image) and the differentiation of adipocytes and osteoblasts were, respectively, detected by Oil Red O staining (middle image) and Alizarin Red staining (right image) (scale bar is 50 µm). Data are representatives of three independent experiments. hASCs, human adipose-derived mesenchymal stem cells; CD, cluster of differentiation; HLA, human leukocyte antigen.

The phenotypes and multiple differentiating capacities were analyzed to identify the characteristics of hASCs used in our experiments. The 4th passage of hASCs was positive for CD90, CD105, and CD73, but negative for CD45, CD34, and HLA-DR (Figure 1a). As expected, hASCs exhibited fibroblastic, adherent characteristics in culture (Figure 1b). Furthermore, hASCs could develop into osteoblasts and adipocytes (Figure 1b) under appropriate conditions. These results indicated that the cells used in our experiments possessed the characteristics of MSCs as described in previous studies [31,32].

### 3.2 Effect of hASCs on body weight gain, food intake, and fat accumulation

**Figure 2:**
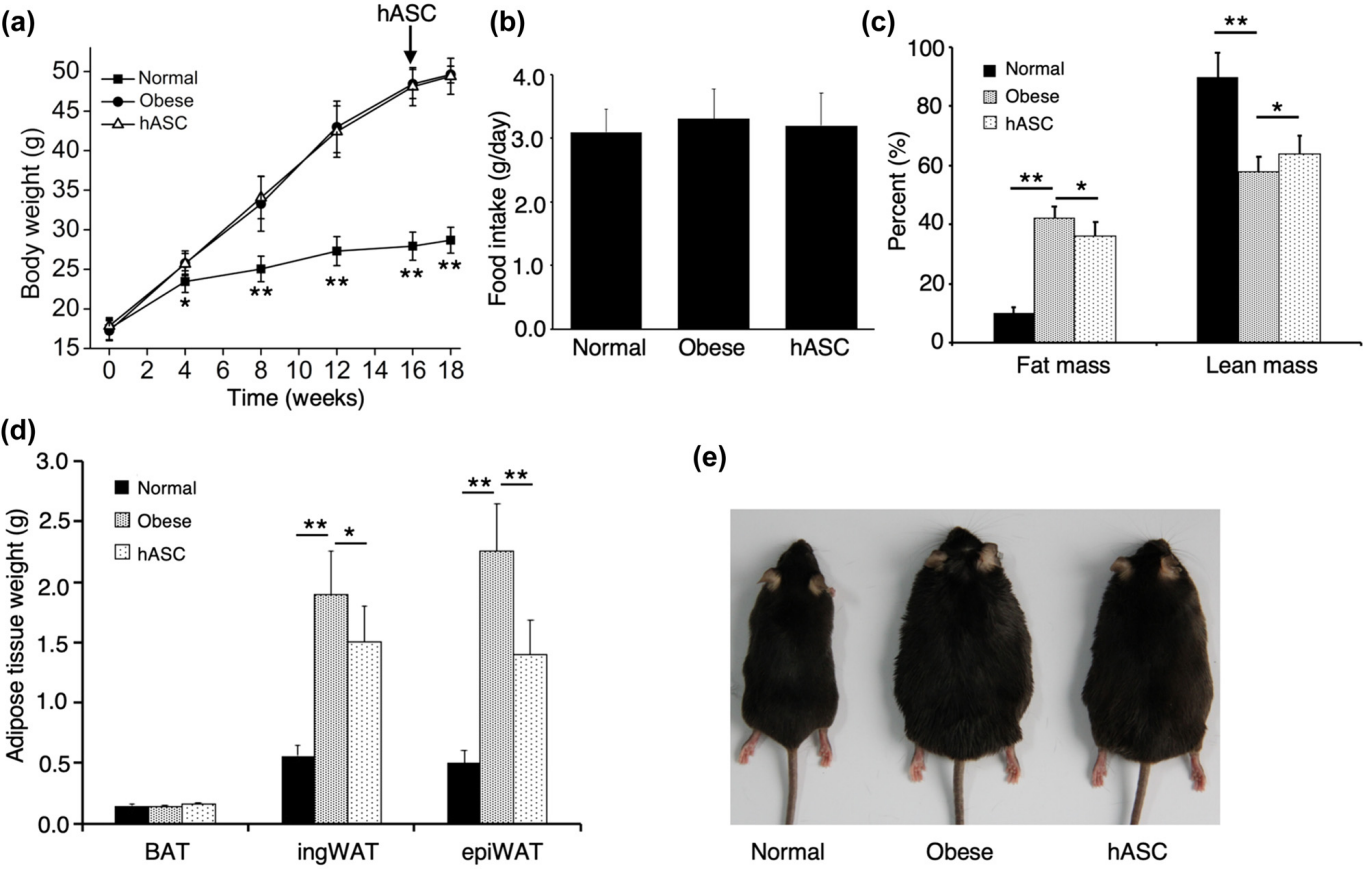
Effect of hASCs on (a) body weight, (b) food intake, (c) fat mass and lean mass, and (d) adipose tissue weight. A representative photograph of the three groups (e). Values are expressed as means ± SD; *n* = 6 mice for each group; *represents *P* < 0.05, **represents *P* < 0.01. BAT, interscapular brown adipose tissue; ingWAT, inguinal subcutaneous adipose tissue; epiWAT, epididymal adipose tissue.

During the 16 weeks of dietary obese mouse model generation period, the average body weight of mice fed with HFD increased more rapidly than the control mice fed with NCD (Figure 2a). As shown in Figure 2a, no obvious difference was observed between the obese group and the hASC group before hASCs infusion. As expected, these two groups had more weight gain than the normal group did. Two weeks after hASCs infusion, though the hASC-treated group didn’t show a significant decrease in body weight (Figure 2a), decreased fat mass and increased lean mass were observed (Figure 2c). Food consumption was almost the same between the three groups (Figure 2b). In accordance with the decreased fat mass, both ingWAT and epiWAT in the hASC group were significantly lower than that in the obese group. Furthermore, a representative photograph of the three groups showed that the obese mice were larger with more greasy hair compared with normal group, which was partially alleviated by hASCs infusion (Figure 2e).

### 3.3 hASCs attenuated serum lipid level and mitigated adipocyte hypertrophy

**Figure 3:**
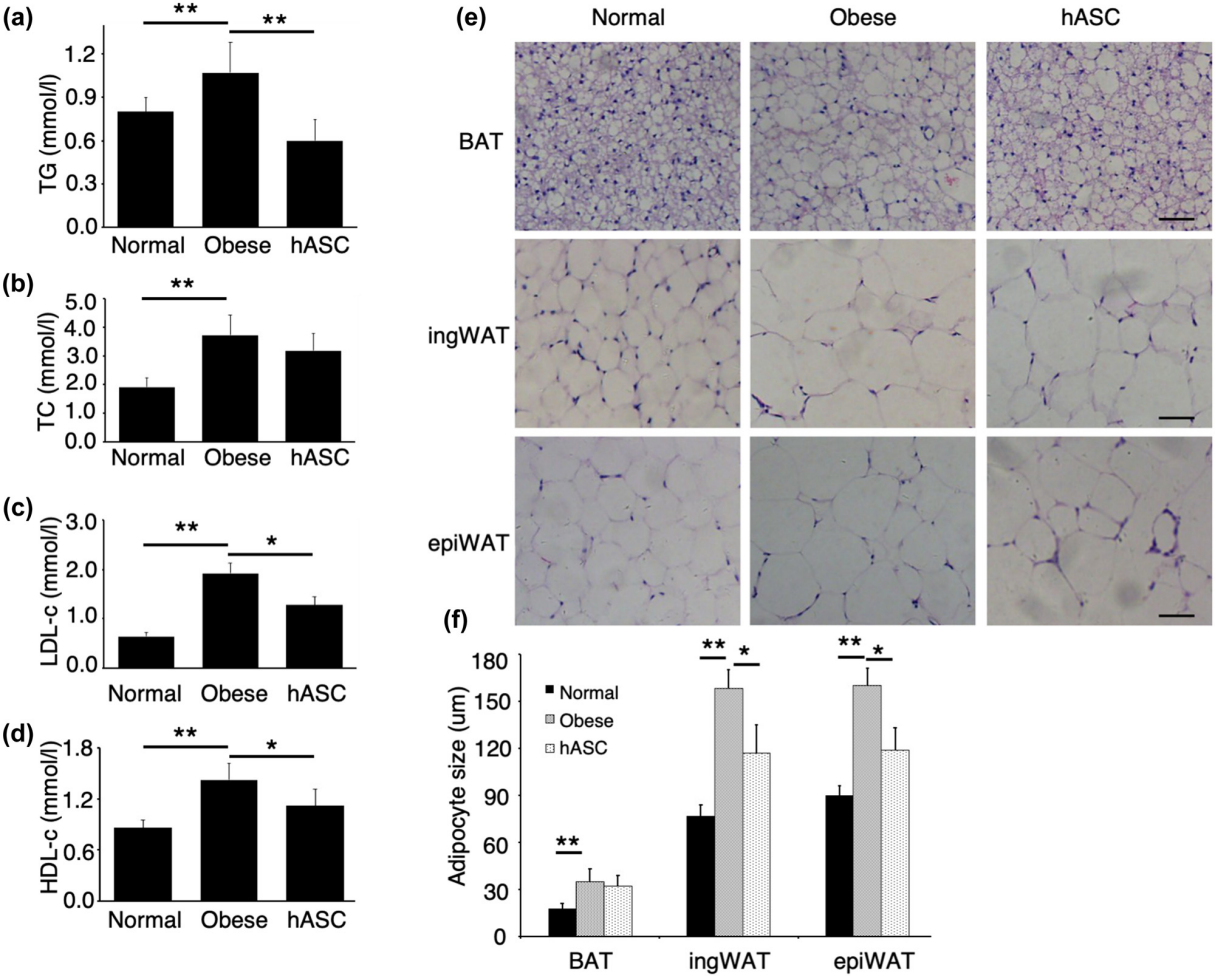
Effects of hASCs on lipid level and adipocyte size. Effects of hASCs on (a) TG, (b) TC, (c) LDL-c, and (d) HDL-c level. (e) HE staining was performed in adipose tissue (the scale bar is 50 µm), (f) The diameter of adipocytes was measured using ImageJ software. TG, triglyceride; TC, total cholesterol; LDL-c, low-density lipoprotein cholesterol; HDL-C, high-density lipoprotein cholesterol. Values are expressed as means ± SD; *n* = 6 mice for each group, *represents *P* ＜ 0.05, **represents *P* < 0.01. The images in *E* are representatives of three independent experiments.

The levels of TG, TC, LDL-c, and HDL-c in the mice fed with HFD were significantly higher than those of the control mice fed with NCD (Figure 3a–d). Intravenous administration of hASCs significantly suppressed the increases in the levels of TG, LDL-c, and HDL-c (Figure 3a, c, and d). The TC level tended to decrease after hASCs infusion, although not significantly (Figure 3b). Compared with normal group, the obese mice showed significant hypertrophic adipocytes in BAT, epiWAT, and ingWAT, whereas administration of hASCs mitigated adipocyte hypertrophy in all the fat depots (Figure 3e). The diameter of adipocytes in HE stained sections was measured using ImageJ software. We found that hASCs infusion resulted in remarkable decrease in adipocyte size, especially in epiWAT and ingWAT (Figure 3f).

### 3.4 Effect of hASCs on energy expenditure

**Figure 4:**
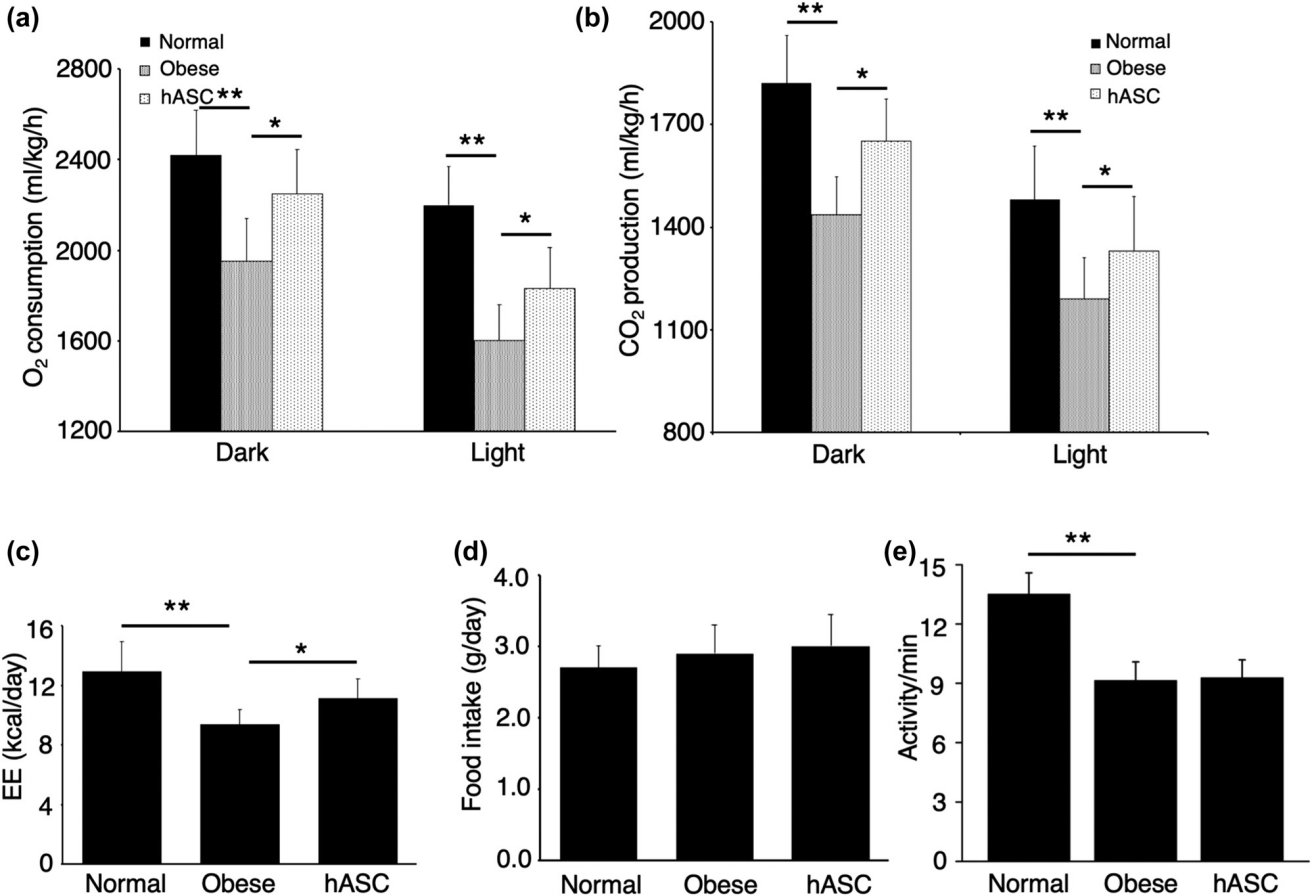
Effect of hASCs on energy expenditure. (a) O_2_ consumption and (b) CO_2_ production in dark and light phases. EE (c), food intake (d), and activity (e) of each group. O_2_, oxygen; CO_2_, carbon dioxide; EE, energy expenditure. Values are expressed as means ± SD; *n* = 6 mice for each group; *represents *P* < 0.05, **represents *P* < 0.01.

As mice were active during dark period, we found that O_2_ consumption and CO_2_ production were higher during the dark period than the light period (Figure 4a and b). Compared with normal group, O_2_ consumption was markedly decreased in obese group, and this was significantly improved by hASCs infusion (Figure 4a). Similarly, CO_2_ production was also remarkably increased by hASCs infusion (Figure 4b). Additionally, as shown in Figure 4c, the obese mice had a lower level of energy expenditure than the normal mice, whereas hASCs infusion increased the energy expenditure with no differences in food intake (Figure 4d) and activity (Figure 4e).

### 3.5 Expression of UCP-1 protein and thermogenic genes

**Figure 5:**
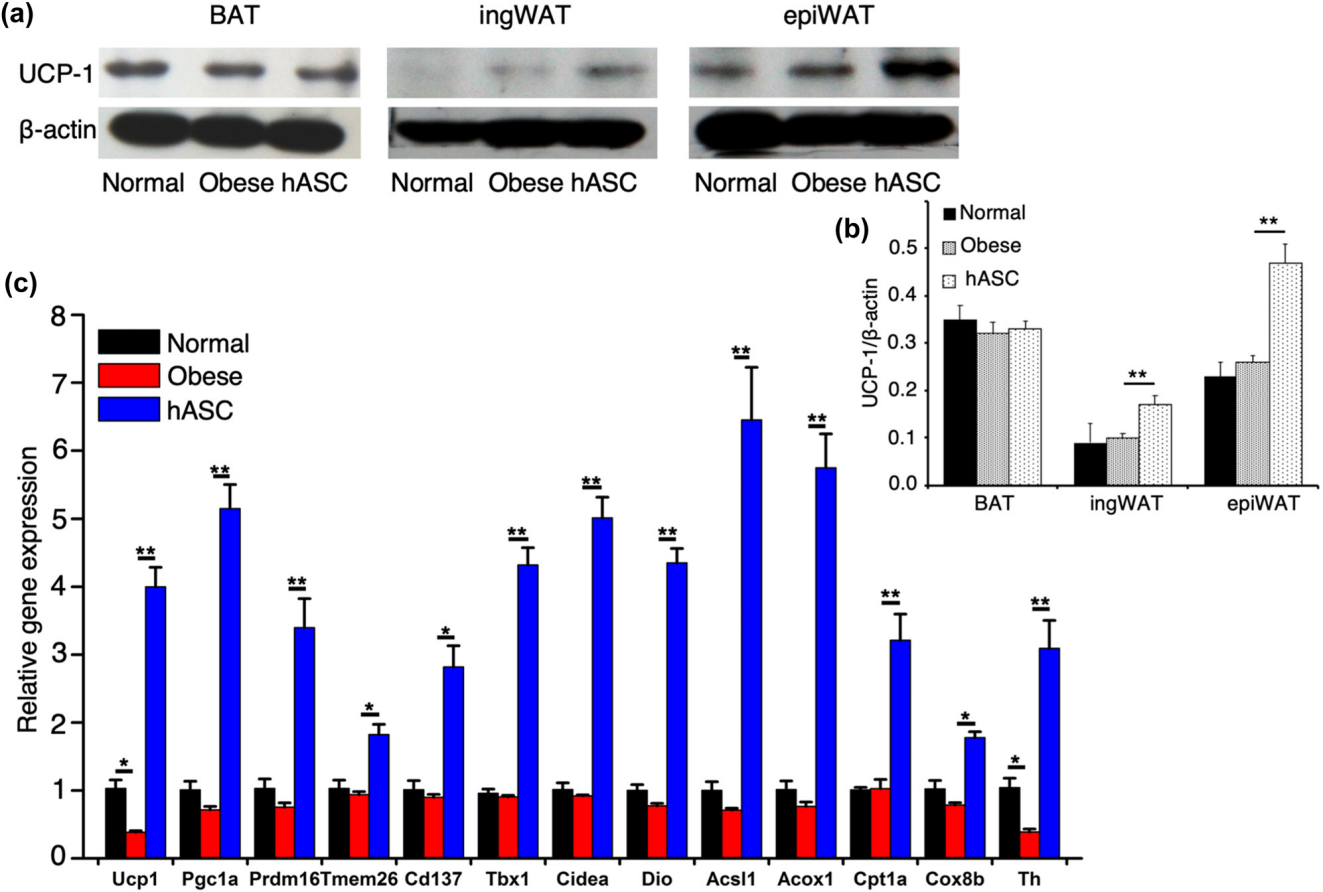
Expression of UCP-1 protein and thermogenic genes. (a) Western blot analysis of UCP-1, representative of three independent experiments. (b) Relative protein level of UCP-1, ratios of UCP-1 to β-actin are quantitated. (c) RT-qPCR analysis of the expression of energy expenditure-related genes in epiWAT, the result is set as 1 in normal group, and the results in the other two groups are expressed relative to normal group. UCP-1, uncoupling protein-1. Values are expressed as means ± SD; *n* = 6 mice for each group, *represents *P* < 0.05, **represents *P* < 0.01.

To further validate that hASCs infusion could improve energy expenditure, we detected the expression of thermogenic-related genes and UCP-1 protein which is required for uncoupled respiration. Protein analysis revealed that UCP-1 expression in the three groups was almost the same in BAT, but was markedly upregulated in ingWAT and epiWAT by hASCs infusion, especially in epiWAT (Figure 5a and b). As the changes were most obvious in epiWAT, we then focused on epiWAT. Consistently, mRNA expression of UCP-1 in epiWAT was also elevated by hASCs infusion. Considering the contribution of adipose tissue browning in energy expenditure, we further detected the gene expression related to brown adipogenic (Pgc1α and Prdm16) and beige fat (Tmem26, Cd137, and Tbx1) markers, mitochondrial activity markers (Cidea, Dio, Acsl1, Acox1, Cpt1a, and Cox8b), and the rate-limiting enzyme (tyrosine hydroxylase, Th) of catecholamine synthesis in epiWAT. Compared with NCD feeding, long-term HFD feeding broadly inhibited the expression of these genes in epiWAT, whereas hASCs infusion significantly reversed their expression (Figure 5c). These data further indicated that hASCs infusion enhanced energy expenditure and potently protected mice from dietary obesity.

### 3.6 Flow cytometry analysis of SVF from epiWAT

**Figure 6:**
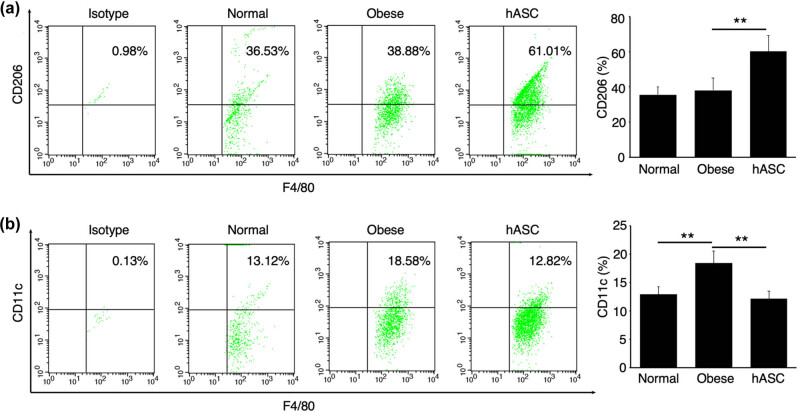
Flow cytometry analysis and quantification of SVF cells for F4/80 with CD206 (a) and CD11c (b), images are representatives of three independent experiments. Values are means ± SD (*n* = 6) of three individual experiments, **represents *P* < 0.01.

In WAT, M2 macrophages have been demonstrated as a major source of catecholamine, which was considered to be crucial in adipose tissue browning. To investigate the effects of hASCs on macrophages phenotype in obese mice, we did flow cytometry analysis of SVF, which was isolated from epiWAT. As shown in Figure 6a, the percentage of F4/80^+^ CD206^+^ M2 macrophages in epiWAT from HFD-fed obese mice significantly increased after hASCs treatment. Meanwhile, Figure 6b showed that the F4/80^+^ CD11c^+^ classically activated (M1) macrophages were markedly reduced by hASCs infusion.

### 3.7 hASCs remodeled macrophage phenotypes

**Figure 7:**
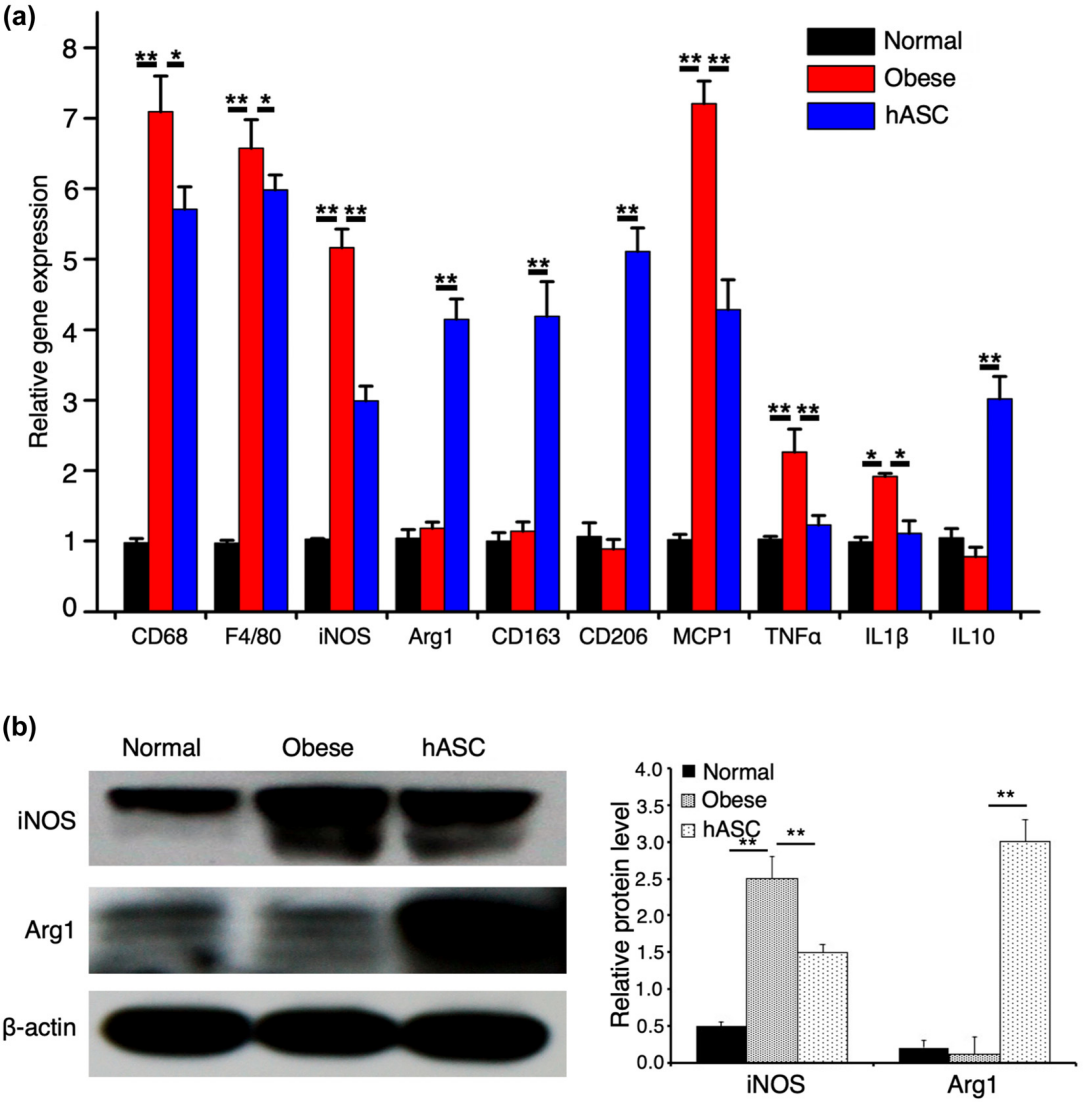
Expression of macrophage phenotypes-related genes and proteins. (a) RT-qPCR analysis of the expression of macrophages phenotypes-related genes in epiWAT, the result is set as 1 in normal group, and the results in the other two groups are expressed relative to normal group. (b) Western blot analysis of iNOS and Arg1, representative of three independent experiments. Relative protein levels are quantified by ratios of iNOS and Arg1 to β-actin. Values are expressed as means ± SD; *n* = 6 mice for each group, *represents *P* < 0.05, **represents *P* < 0.01.

To further verify the effect of hASCs on adipose tissue macrophages, we analyzed the expression of M1 and M2 macrophages-related genes in SVF from epiWAT. The remarkably elevated expression of CD68 and F4/80 in obese mice demonstrated increased infiltration of macrophages in WAT during dietary obesity, which was suppressed by hASCs infusion (Figure 7a). The mRNA levels of iNOS, MCP1, TNFα, and IL1β, which were M1 phenotypes induced by obesity, were significantly decreased in SVF from HFD-fed mice treated with hASCs (Figure 7a). And, also hASCs infusion led to a dramatic increase in mRNA levels of Arg1, CD206, CD163, and IL10, representatives of M2 phenotypes in SVF (Figure 7a). Consistently, western blot analysis revealed that the protein level of iNOS induced by HFD feeding was obviously alleviated by hASCs infusion, while the protein level of Arg1 was elevated (Figure 7b). All these data indicated that hASCs infusion could increase M2 macrophages in epiWAT.

## 4 Discussion

Obesity and its related diseases such as cancer, hypertension, and diabetes have threatened the public health [33]. Traditional anti-obesity methods such as dieting and exercise challenge the obese patients and the effect is far from being satisfactory. Thus, it’s urgent to figure out effective interventions to deal with obesity. Studies have found that obesity is a result of accumulated WATs, which possess the function of storing energy and eventually bring adverse effects to health [34]. However, BAT, the other type of adipose tissue, has been confirmed to consume energy [10]. And it has also been demonstrated that increasing the amount of brown-like adipose tissue can promote energy expenditure and limit obesity [12,16]. Recently, increasing the secretion of catecholamine [13,15,16,17] and the modification of pre-adipocyte’s differentiation-related genes [35,36] have been widely studied to promote adipose tissue browning.

Obesity, considered as a chronic inflammatory disease, is accompanied with excess accumulation of adipose tissue [37]. This adipose tissue accumulation can lead to local hypoxia and infiltration of immune cells such as neutrophil, macrophages, lymphocytes, etc. [38]. Thus, adipose tissue is not only an energy storage organ, but also the main source of inflammation, with the elevation secretion of inflammatory factors such as TNFα and IL1β, and adipocytokines like omentin [39] and neuregulin-4 [40]. Furthermore, this chronic inflammation during obesity is now considered as the main initiator of obesity-related disorders, such as type 2 diabetes [41], frailty [42], and cardiac conditions [43]. The macrophages infiltrated in adipose tissue are majorly divided into two types, classically activated macrophages (M1) and alternatively activated macrophages (M2) [44]. M1 macrophages, also named inflammatory macrophages, mainly secrete inflammatory molecules such as TNFα and IL1β, while M2 macrophages are featured with anti-inflammatory molecule IL10. Previous studies have shown that the macrophages accumulated during obesity are majorly M1 macrophages [45]. Promoting M2 macrophage polarization facilitates WAT browning [15,16] and alleviates insulin resistance [37,46]. What is more, our previous study has confirmed that intravenously infused MSCs can increase M2 macrophages in adipose tissue [25]. Therefore, in this study we explored the effect of MSCs on diet-induced obesity and explored the underlying mechanism.

The MSCs in this experiment, which was isolated from the obese patient’s visceral adipose tissue, possessed the internationally defined characteristics as expected [19]. We demonstrated that hASCs infusion could remarkably decrease the percent of fat mass and increase the percent of lean mass. And also the histologic analysis showed that hASCs infusion significantly reduced the adipocyte hypertrophy induced by HFD feeding, in accordance with increased O_2_ consumption, CO_2_ production, and energy expenditure. What is more, no differences in food intake were observed between obese group and hASCs group, indicating that the anti-obesity effects were not due to the anorectic effect caused by hASCs infusion. Consistently, Figure 2e showed that the larger shape in obese group was alleviated by hASCs infusion. The unchanged weight may be due to the short experimental time.

Previous studies have demonstrated that UCP-1 protein is required for uncoupled respiration and is responsible for the beneficial effect of BAT [47]. This study found that hASCs infusion upregulated the expression of UCP-1 and the energy metabolism-related genes in WAT. Consistently, the histologic analysis revealed that the adipocyte size was reduced by hASCs infusion. Altogether, the upregulation of UCP-1, brown adipogenic, and beige fat markers, along with the decreased adipocyte size, suggested the adipose tissue browning, which reconfirmed the enhanced energy expenditure.

To further figure out the mechanism of hASCs on obesity, we analyzed the macrophages phenotypes in adipose tissue. The results of flow cytometry analysis, western blot analysis, and RT-qPCR revealed that hASCs infusion increased M2 macrophages in adipose tissue. Previous studies have demonstrated that M2 macrophages express tyrosine hydroxylase that catalyzes the production of catecholamine, thereby driving WAT browning [15,16]. Thus, we might conclude that hASCs attenuate obesity through polarizing M2 macrophages, which was in agreement with prevenient study [48]. However, to further emphasize the importance of macrophages in mediating the effect of hASCs, future studies need to figure out the molecular mechanism of polarizing M2 macrophages and introduce transgenic mice. Additionally, there are still some unanswered questions that may limit the use of hASCs in clinical translation. First, we need to figure out the distribution of intravenously infused hASCs *in vivo* and how these cells exert immunomodulatory function. Second, the appropriate number of hASCs needs to be sought to exert the best therapeutic effect in clinic. Third, the long-term efficacy and safety of hASCs on obesity need further evaluation.

In conclusion, the findings of the study demonstrated that hASCs infusion decreased fat mass and suppressed adipocyte hypertrophy in the HFD-induced obese mice model. And this effect was due to the enhanced energy expenditure with the upregulation of UCP-1 protein and thermogenic genes. Furthermore, we found that hASCs infusion elevated the amount of M2 macrophages in adipose tissue, which partially explained the effect of hASCs on obesity. As we know, hASCs are easily obtained and amplified from adipose tissue. Moreover, hASCs are low immunogenicity. These characteristics determined that hASCs might be a promising therapy for obesity.
